# Six-Strand Flexor Pollicis Longus Tendon Repairs With and Without Circumferential Sutures: A Multicenter Study

**DOI:** 10.1177/15589447211057295

**Published:** 2022-01-07

**Authors:** Géraldine Lautenbach, Marco Guidi, Bernadette Tobler-Ammann, Vera Beckmann-Fries, Elisabeth Oberfeld, Lorena Schrepfer, Sebastian Hediger, Alexandre Kaempfen, Esther Vögelin, Maurizio Calcagni

**Affiliations:** 1 University Hospital Zurich, Switzerland; 2 Inselspital, Bern, Switzerland; 3 University Hospital Basel, Switzerland

**Keywords:** early active motion, circumferential suture, flexor pollicis longus tendon, primary flexor tendon repair, without circumferential suture

## Abstract

**Background:**

The purpose of this study is to assess outcomes in flexor pollicis longus tendon repairs with 6-strand core sutures with and without circumferential sutures.

**Methods:**

A 6-strand core suture technique with and without circumferential sutures was used. Thirty-three patients were summarized in the C group (circumferential group) and 16 patients in the NC group (non-circumferential group). After the surgery, the wrist was stabilized with a dorsal blocking splint and a controlled early active motion protocol was applied. At weeks 6, 13, and 26 data on demographics, type of injury, surgery, postoperative rehabilitation, complications such as re-rupture and the following outcome measurements were collected: range of motion and its recovery according to the Tang criteria, Kapandji score, thumb and hand strengths, Disabilities of the Arm, Shoulder and Hand score, and satisfaction.

**Results:**

There were no significant differences in range of motion and strength between the 2 treatment groups. In both groups, the outcome measurements increased over time and they expressed similar satisfaction with the surgical treatment. In 4 patients of the C group tendon repair ruptured and in 1 patient of the NC group.

**Conclusions:**

Six-strand repair technique is an effective procedure to assure early active motion after flexor pollicis longus tendon injuries and good results can also be achieved by omitting the circumferential suture.

## Introduction

Recovery of good function after flexor pollicis longus (FPL) tendon injuries is crucial to regain optimal function of the hand. To achieve this, a robust tendon repair is a prerequisite for early mobilization. Two-strand suture methods are considered not enough to allow an early active digital motion.^1-5^ For this reason, new suture techniques were developed in the last 3 decades, to increase primary repair strength and gapping resistance. Good functional results for 6-strand core sutures of flexor tendon lacerations were reported.^6-9^ Some authors began to omit the circumferential suture to reduce the volume of the repair and the amount of suture material and therefore improve the tendon gliding, without jeopardizing the strength of the repair.^6^ In literature only few reports on outcomes in FPL tendon repairs without circumferential sutures are described.^10^ The purpose of this study was to assess outcomes in FPL tendon repairs with a 6-strand core sutures with and without circumferential sutures.

## Materials and Methods

### Inclusion and Exclusion Criteria

**Figure 1.**
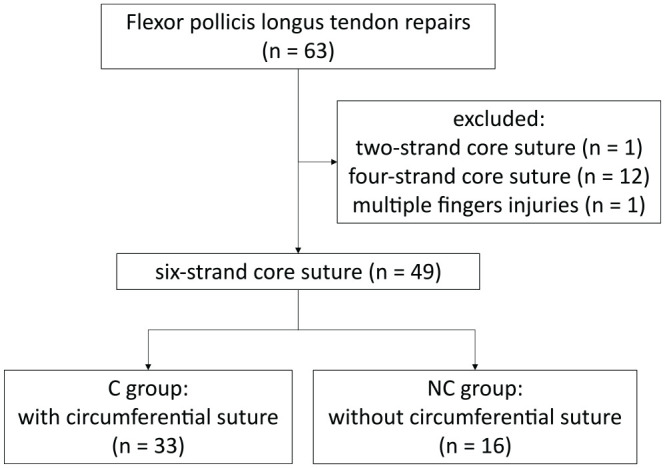
Study flow-chart. C group = circumferential group; NC group = non-circumferential group.

From January 2014 to December 2020, 63 patients with FPL tendon ruptures were consecutively recruited at the involved centers (Figure 1). Inclusion criteria were age between 18 and 75 years, flexor tendon injury over 50% in zone I-III, surgery and completed hand therapy at 1 of the 3 centers, controlled active motion (CAM) protocol, and informed consent. Exclusion criteria were core sutures with other than 6 strands and multiple finger injuries. In total, 14 patients were excluded because of 2-strand core sutures (n = 1), 4-strand core sutures (n = 12), and multiple finger injuries (n = 1).

### Demographics

All patients, whose tendons were repaired with a circumferential suture were in the circumferential group (C group, n = 33), and the patients, whose tendons were repaired without a circumferential suture were in the non-circumferential group (NC group, n = 16).

Mean age was 36 years in the C group and 33 years in the NC group. In the C group were 18 men and 15 women compared to 12 men and 4 women in the NC group (Table 1). The distribution in blue and white collar workers were similar in both groups (C group: 15 blue, 14 white, 4 unknown. NC group: 8 blue, 7 white, 1 unknown).

**Table 1.** Demographics, Injury, Surgery and Hand Therapy Characteristics.

Characteristics	C group	NC group
Demographics		
Number of patients (men/women)	33 (18/15)	16 (12/4)
Mean (SD) age and range at injury (years)	36 (14), 18-67	33 (10) 20-63
Collar worker (blue/white/unknown)	15/14/4	8/7/1
Injuries		
Dominant/non-dominant/unknown	9/23/1	7/9/0
Zone I/II/III	11/19/3	4/11/1
Clean cut/mild crush/moderate crush	28/1/4	14/2/0
Complete/incomplete tendon laceration	32/1	14/2
Concomitant injuries		
Nerve	20	10
Joint	6	1
A1/oblique/A2 pulley	1/4/1	0/2/0
Muscle in zone III	1	1
Surgery		
Mean (SD) time and range from injury to tendon repair (days)	4 (6), 0-28	3 (3), 0-8
Pulley repair	6	0
Pulley venting	9	9
Hand therapy		
Mean (SD) duration and range (weeks)	20 (8), 3-40	16 (5), 5-23
Mean (SD) number and range of sessions	17 (5), 5-30	18 (6), 10-29
Long dorsal blocking splint	33	16
IP extension splint	8	7

*Note.* C group = circumferential group; NC group = non-circumferential group; IP = interphalangeal joint; SD = standard deviation.

### Injuries

The non-dominant hand was injured in 23 patients in the C group and 9 in the NC group, and the dominant in 9 patients in the C group and 7 in the NC group. The most frequent mechanism of injury was a clean cut in 28 patients in the C group and 14 in the NC group, followed by a mild crush in 1 patient in the C group and 2 patients in the NC group and a moderate crush in 4 patients (all C group). Most lesions were located in zone II (19 in C group and 11 in NC group). Almost all patients had a complete tendon laceration (32 in C group and 14 in NC group). The most frequent concomitant injured structure was a nerve in 20 patients in C group and in 10 patients in NC group, followed by pulley and joint injuries.

### FPL Tendon Repairs

The FPL tendon repair was carried out under plexus anesthesia in 30 patients (22 C group and 8 NC group), under general anesthesia in 11 patients (8 C group and 3 NC group) and under local anesthesia in 8 patients (3 C group and 5 NC group). The lacerated FPL tendons were repaired with 6-strand core sutures. The surgeons aimed for a slight bulkiness at the repair site with the core sutures. Afterwards a simple running circumferential suture was added in the C group, when the hand surgeon decided to improve the matching of the 2 parts of the tendon or in case of a persisting gap. Six patients had a pulley repair (all in C group) and 18 needed a pulley venting (9 in C group, 9 in NC group). Mean time from the accident to primary surgery was 4 days in C group and 3 days in NC group.

### Hand Therapy Intervention

Three to 5 days postsurgery, a dorsal blocking splint was adapted by a specialized hand therapist. The therapists aimed for a splint with the wrist positioned in 10° extension and 10° ulnar deviation. The thumb was held in moderate palmar abduction, the metacarpophalangeal (MCP) joint in 10 to 20° flexion and the interphalangeal (IP) joint in neutral position. The splint was worn day and night for 5 weeks and only at night for another 3 weeks. Exercises were instructed based on the modified CAM protocol.^11^ Active IP joint motion was initiated within the first 7 days after surgery. The amount of active movement increased weekly: active thumb flexion to the base of the ring finger (2nd postoperative week) and to the base of the little finger (from 3rd postoperative week onwards). The full passive range of motion (pROM; flexion to the base of the little finger) was advocated from the first day in hand therapy, and patients were prompted to always passively mobilize the joints and then start with the active exercises. Light activities of daily living were initiated 6 to 8 weeks postoperatively, and full use was permitted after 12 weeks.

### Assessment of Outcomes

The primary outcome measure was thumb range of motion being assessed at weeks 6, 13, and 26. The total active and passive ranges of motion (TAM and TPM) were measured using the sum of active range of motion (aROM) or pROM of the IP and MCP joint. For the contralateral thumb the TAM was collected at week 6. Furthermore, return of range of motion was graded according to the Tang criteria of 2007.^12^ Thumb opposition/flexion was assessed with the Kapandji score.

Secondary outcome measurements were assessed at weeks 13 and 26, including strength, level of disability, and satisfaction. Hand grip strength was measured using a Jamar Hydraulic Hand Dynamometer. Thumb key pinch strength was measured using a Jamar Pinch Gauges Dynamometer. Hand grip and thumb key pinch were also assessed for the contralateral thumb at all time points. To assess the level of self-perceived disability and symptoms of the upper extremity, the Disabilities of the Arm, Shoulder and Hand (DASH) questionnaire was used.^13^ It consists of a 30-item scale and 2 optional 4-item scales for work and sports/music performance, where a higher score indicates greater disability. To document satisfaction with the injured hand, a Single Assessment Numeric Evaluation was used.^14^ A higher score indicates higher satisfaction.

Furthermore, it was noted whether the patient was able to return to work. And the following adverse events were noted and treated accordingly: allodynia, tendon adhesion, ossification, infection, complex regional pain syndrome, and re-rupture. All patients with re-ruptures were not included in the primary and secondary outcome measurements, as assessments of the functional outcomes were not possible.

### Statistical Analysis

Data were summarized as mean and standard deviations (SD), and median and ranges. For all analyses, non-parametric tests were applied due to the small sample size. Differences over time in TAM, strength, Kapandji, satisfaction, and DASH scores were analyzed per group using the Wilcoxon test. Differences between the 2 groups for TAM, strength Kapandji, satisfaction, and DASH scores were analyzed with the Mann-Whitney-U test. Effect sizes were calculated using Cohen’s classification of small (*r* = 0.10), medium (*r* = 0.30), and large (*r* = 0.50) effects.^15^ Level of significance was set at *P* < 0.05. No corrections were made for missing values.

### Ethics

The study was carried out in accordance with the standards of the local ethical committee on human experimentation (institutional and national) and with the Helsinki Declaration. Informed consent was obtained from all patients before entering the study.

## Results

### Primary Outcome Measurements

**Figure 2.**
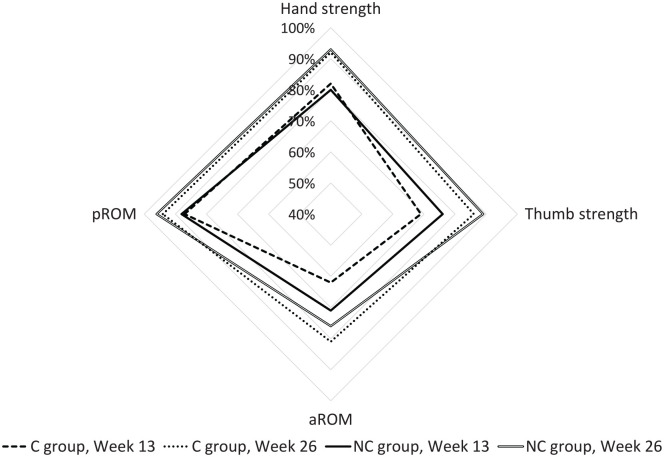
Percentage of recovery over time in aROM, pROM, hand, and thumb strength with the results of the contralateral thumb as 100%. *Note.* C group = circumferential group; NC group = non-circumferential group; aROM = active range of motion; pROM = passive range of motion.

**Figure 3.**
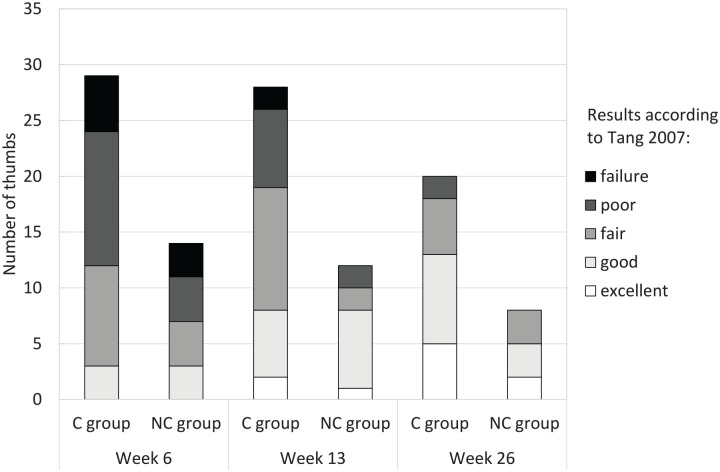
Number of thumbs graded according to Tang 2007 at weeks 6, 13, and 26. C group = circumferential group; NC group = non-circumferential group.

Mean aROM and pROM values and Kapandji scores at week 6, 13, and 26 in both groups are presented in Table 2 and Supplementary Table S1. The percentage of recovery over time is graphically displayed in Figure 2 and the recovery of motion graded according to Tang^12^ in Figure 3.

**Table 2.** Range of Motion Scores for C and NC Group at Weeks 6, 13 and 26.

Examination	Week 6		Week 13		Week 26	
	C group	NC group	C group	NC group	C group	NC group
	Mean (SD), range	Mean (SD), range	Mean (SD), range	Mean (SD), range	Mean (SD), range	Mean (SD), range
TAM (°)	78 (25), 20-130	79 (32), 20-120	106 (34), 40-175	119 (26), 85-175	137 (37), 55-215	128 (19), 90-155
TPM (°)	133 (19), 90-170	132 (28), 80-170	150 (28), 105-215	150 (17), 130-175	165 (31), 105-220	163 (16), 150-185
Kapandji score	8 (1), 6-10	8 (1), 5-10	9 (1), 7-10	9 (1), 5-10	10 (1), 8-10	9 (2), 5-10

*Note.* C group = circumferential group; NC group = non-circumferential group; TAM = total active range of motion; TPM = total passive range of motion; SD = standard deviation; ° = degree.

Correlations between TAM scores in the C and NC groups were statistically significant (*P* = 0.00) with large effect sizes (*r* = 0.79 and 0.88) for measurement points between week 6 to 13. For measurements between week 13 and week 26 significant differences (*P* = 0.01, *r* = 0.54) were measured in the C group and no significant differences (*P* = 0.58, *r* = 0.20) in the NC group. For measurements between the injured and uninjured thumb, significant differences (*P* = 0.00 and 0.03, *r* = 0.66 and 0.89) were measured in both groups in week 26.

For TPM scores, no statistically significant differences (*P* = 0.06-0.35) with large effect sizes (*r* = 0.35-0.62) were measured between week 6 and week 13 in the NC group and between week 13 and week 26 in both groups. In the C group, a statistically significant difference (*P* = 0.00) was measured between week 6 and week 13.

Statistically significant differences were measured for Kapandji scores in both groups between week 6 and week 13 (*P* = 0.00) with large effect size (*r* = 0.70 and 0.88) and no statistical significances between week 13 and week 26 (*P* = 0.08 and 1.00, *r* = 0.36 and 0.00). At week 26, the difference of the Kapandji scores between the injured and uninjured thumbs was not statistically significant (*P* = 0.27, *r* = 0.25) in the C group, but significant (*P* = 0.03, *r* = 0.80) in the NC group.

The TAM, TPM, and Kapandji scores between the C and NC groups were not statistically significant (*P* = 0.11-0.94), with small effect sizes in all (*r* = 0.01 to 0.21) but 1 time point. Medium effect sizes were measured at week 26 for the Kapandji scores (*r* = 0.37) between both groups, favoring the C group.

### Secondary Outcome Measurements

**Figure 4.**
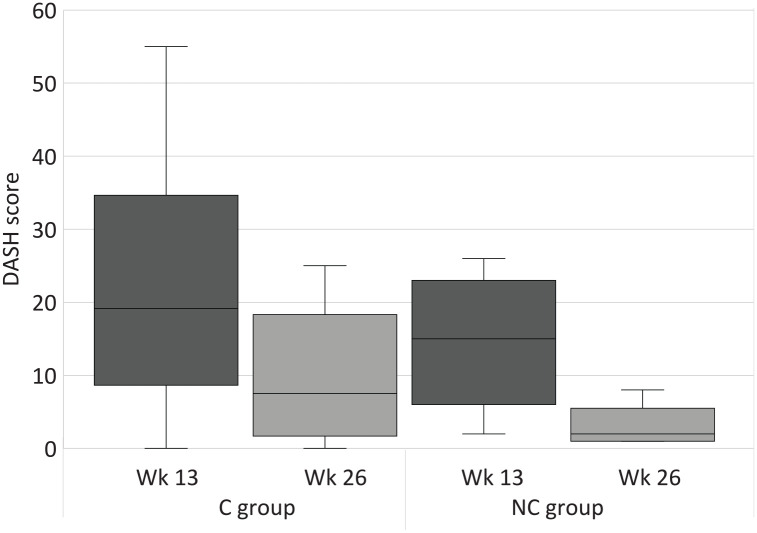
DASH scores at week 13 and week 26. Wk = week; C group = circumferential group; NC group = non-circumferential group; DASH = Disabilities of the Arm, Shoulder and Hand.

**Figure 5.**
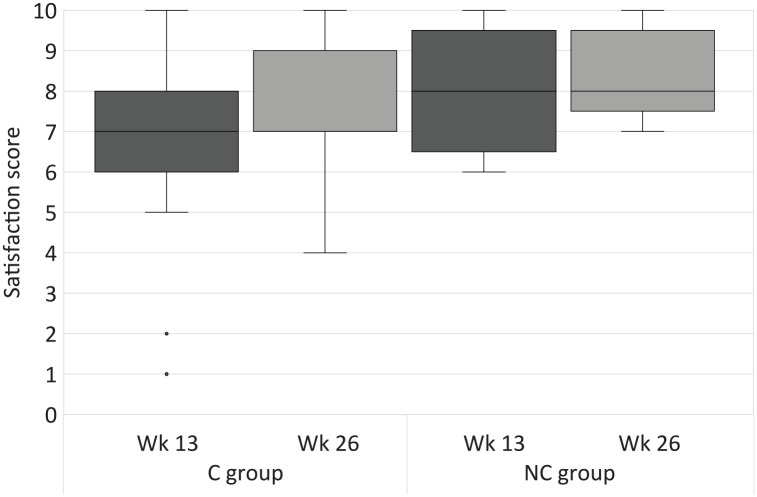
Satisfaction scores at week 13 and week 26. Wk = week; C group = circumferential group; NC group = non-circumferential group.

Results for the strength, DASH, and patient satisfaction scores are summarized in Supplementary Table S2, and changes over time are graphically displayed in Figures 2, 4 and 5 for weeks 13 and 26. Hand strength was measured not statistically significant (*P* = 0.39 and 0.21, *r* = 0.19 and 0.45) between week 13 and week 26 in both groups. Thumb strength was measured statistically significant between week 13 and week 26 in the C group (*P* = 0.01, *r* = 0.57) and not statistically significant with large effect sizes (*P* = 0.08, *r* = 0.73) in the NC group. Patients in the C group significantly improved their physical function and satisfaction over time (*P* = 0.00 to 0.01) with large effect sizes (*r* = 0.61). The improvements in the NC group were not statistically significant (*P* = 0.07 and 0.93, *r* = 0.91 and 0.03). Comparisons of hand and thumb strengths with the contralateral side were not statistically significant at week 26 (*P* = 0.33 to 1.00, *r* = 0.00 to 0.22).

There were no statistically significant differences in all secondary outcome measures between the C and NC groups at any time point (*P* = 0.07 to 1.00) with correspondingly small to medium effect sizes (*r* = 0.00 to 0.31). Medium effect sizes were measured for satisfaction (*r* = 0.31) in week 13, favoring the NC group.

At week 13, loss of mobility was the main reason for dissatisfaction with the thumb (C group: n = 11, NC group: n = 3), followed by loss of strength (n = 3) and pain (n = 3) in the C group and sensory deficits in the NC group (n = 2).

At week 26, loss of mobility (n = 5) and sensory deficits (n = 4) were mentioned as disturbing in the C group and loss of strength (n = 1) and loss of strength (n = 1) in the NC group.

### Adverse Events and Return to Work

In the C group, 4 tendon repairs ruptured. One of these patients had an infection as a second adverse event. Three patients had tendon adhesions, and another patient reported allodynia. In the NC group the tendon repair of 1 patient ruptured, and no allodynia, tendon adhesions, or infections were reported. In none of the groups complex regional pain syndromes or ossifications were noted as adverse events.

All patients with re-ruptures were male, and most of them were blue collar workers with a mean age of 34 years old. The primary surgery was carried out in a mean of 2 days, and the secondary surgery in a mean of 24 days. One patient decided against surgery. He was self-employed, and the rupture was on the non-dominant hand.

Twenty-seven out of 33 patients in the C group and 14 out of 16 patients in the NC group returned to work after the tendon repairs. The mean time of sick leave was 15 weeks in the C group and 10 weeks in the NC group.

## Discussion

Most patients in the C group achieved good to excellent results according to the Tang criteria in week 26. The study by Pan et al^7^ showed similar results. The authors analyzed 6-strand FPL tendon repairs with circumferential sutures in 34 patients and obtained good to excellent results in 30 patients after a mean of 14 months. Also in Moriya et al’s^16^ study, 11 out of 17 patients obtained good to excellent results after a mean of 8 months. In a biomechanical study in a canine model by Putterman et al^17^ the yield (195.2 vs 91.4 N), peak (214.3 vs 91.9 N), and failure force (214.3 vs 91.9 N) was increased more than twofold in tendon repairs with circumferential sutures compared to tendon repairs without circumferential sutures.^17^ But because the level of forces that canine flexor tendons have to withstand is not directly comparable to those in human flexor tendons, the significance of this result for FPL tendon repairs is limited. Originally, the circumferential suture was considered important in smoothening the repair site.^18^ And therefore, it improved the tendon gliding under the pulley.^19^ In the course of time, the circumferential suture developed its relevance in increasing the strength of the repair and the resistance to gap formation.^20^ Moriya et al^20^ concluded that circumferential sutures are necessary, because during the first 2 to 3 weeks after surgery only the primary surgical repair strength is holding the tendon ends together.

In contrast, Giesen et al^6^ argued, that the addition of a circumferential suture may impair the tendon gliding under the pulleys. The authors examined 50 FPL tendon repairs without circumferential sutures and reported in 41 patients excellent and good results according to the Buck-Gramcko criteria.^21^ Almost a decade later, Giesen et al^22^ analyzed 5 more patients with a 6-strand FPL tendon repair without a circumferential suture. Four of the 5 patients achieved good to excellent results after a mean of 9 months. The present study showed a similar outcome with 5 out of 8 patients with good or excellent results at week 26.

That the omission of a circumferential suture may not bring a disadvantage regarding the gapping formation was shown in a sonographic investigation on FDP tendon repairs without circumferential sutures by Reissner et al.^23^ In none of the 10 patients gapping was observed during active movement from 3 days to 12 months postoperatively.^23^ Furthermore, the omission of a circumferential suture reduces the interference with the vascularity of the FPL tendon and therefore may improve tendon healing.^24^

Tendon re-ruptures represent a possible complication after every tendon repair. In the current study, FPL tendon repairs failed in 4 patients in the C group and in 1 patient in the NC group. In comparison, no ruptures occurred in 55 patients with 6-strand FPL tendon repairs without circumferential sutures in the studies proposed by Giesen and colleagues.^6,22^ Pan et al^7^ did not report any ruptures in 34 FPL tendon repairs treated with 6-strand core and circumferential sutures. But Moriya et al^16^ did report 3 ruptures in 17 FPL tendon repairs explained this result with the fact that these repairs were done by inexperienced surgeons.

It is unlikely that the use of a controlled active mobilization regimen instead of an early passive motion could be the reason for the higher rupture rate in this study, as studies comparing active and passive protocols found no significant difference in the risk for ruptures.^25,26^

In the present study, only patients in the C group (n = 3) needed a tenolysis after a mean of 48 weeks. In this context, duration of hand therapy was longer in the C group than in the NC group (20 vs 16 weeks). Since only patients from the C group showed this complication, the circumferential suture could be responsible for the formation of tendon adhesions. Kubota et al^27^ showed that with an increasing number of strands of the circumferential suture in FDP tendon repairs, the holding power increases, but at the same time leads to increased friction. This is associated with a higher risk for adhesions.^28^

The present research has some strengths, including a follow-up and a standardized rehabilitation protocol. There are also certain limitations. The samples of the 2 groups were unbalanced due to surgical differences in the involved centers (eg, in 1 hospital a circumferential suture was always added). A larger and randomized cohort study might be a viable solution, increasing the power and validity of the findings.

## Conclusion

In conclusion, in both groups the 6-strand core suture with or without circumferential suture allowed for a good functional recovery in most patients, with a low complication rate. Only small differences were observed between the 2 groups, suggesting that omitting the circumferential suture may not have clinical disadvantages. A trend towards a higher need for tenolysis was observed in patients where a circumferential suture was used, suggesting that this should only be used in cases where tendon gaps are obvious, or the stumps are too irregular to allow unrestricted gliding under the pulleys.

## Supplemental Material

sj-docx-1-han-10.1177_15589447211057295 – Supplemental material for Six-Strand Flexor Pollicis Longus Tendon Repairs With and Without Circumferential Sutures: A Multicenter Study Click here for additional data file. Supplemental material, sj-docx-1-han-10.1177_15589447211057295 for Six-Strand Flexor Pollicis Longus Tendon Repairs With and Without Circumferential Sutures: A Multicenter Study by Géraldine Lautenbach, Marco Guidi, Bernadette Tobler-Ammann, Vera Beckmann-Fries, Elisabeth Oberfeld, Lorena Schrepfer, Sebastian Hediger, Alexandre Kaempfen, Esther Vögelin and Maurizio Calcagni in HAND

sj-docx-2-han-10.1177_15589447211057295 – Supplemental material for Six-Strand Flexor Pollicis Longus Tendon Repairs With and Without Circumferential Sutures: A Multicenter Study Click here for additional data file. Supplemental material, sj-docx-2-han-10.1177_15589447211057295 for Six-Strand Flexor Pollicis Longus Tendon Repairs With and Without Circumferential Sutures: A Multicenter Study by Géraldine Lautenbach, Marco Guidi, Bernadette Tobler-Ammann, Vera Beckmann-Fries, Elisabeth Oberfeld, Lorena Schrepfer, Sebastian Hediger, Alexandre Kaempfen, Esther Vögelin and Maurizio Calcagni in HAND
