# Supplementary material for: Six-Strand Flexor Pollicis Longus Tendon Repairs With and Without Circumferential Sutures: A Multicenter Study
Source: Hand (N Y). 2022 Jan 7;18(5):811–9. doi: 10.1177/15589447211057295 (PMC10336814; doi:10.1177/15589447211057295)
Supplement: sj-docx-1-han-10.1177_15589447211057295 – Supplemental material for Six-Strand Flexor Pollicis Longus Tendon Repairs With and Without Circumferential Sutures: A Multicenter Study [file sj-docx-1-han-10.1177_15589447211057295.docx]

| Supplementary Table S1. Details to range of motion scores at weeks 6, 13 and 26 | | | | | | |
| --- | --- | --- | --- | --- | --- | --- |
|  | Week 6 | | Week 13 | | Week 26 | |
| Examination or test | C group | NC group | C group | NC group | C group | NC group |
|  | Mean (SD), range (°) | Mean (SD), range (°) | Mean (SD), range (°) | Mean (SD),  range (°) | Mean (SD),  range (°) | Mean (SD),  range (°) |
| aROM of the injured thumb | | | | | | |
| MCP joint | 48 (13), 20-75 | 50 (19), 10-75 | 65 (15), 40-105 | 66 (18), 35-95 | 70 (16), 45-105 | 69 (17), 50-95 |
| IP joint | 30 (17), 0-75 | 29 (19), 0-55 | 41 (26), 0-110 | 53 (27), 15-95 | 67 (32), 0-130 | 59 (25), 20-105 |
| pROM of the injured thumb | | | | | | |
| MCP joint | 67 (11), 45-90 | 65 (13), 50-95 | 77 (15), 50-115 | 72 (18), 50-100 | 80 (15), 60-115 | 75 (25), 50-100 |
| IP joint | 66 (14), 35-95 | 73 (18), 45-100 | 73 (20), 35-135 | 78 (17), 55-105 | 85 (22), 40-140 | 88 (14), 75-105 |
| aROM of the uninjured thumb | | | | | | |
| MCP joint | 70 (15), 40-100 | 64 (14), 40-90 |  |  |  |  |
| IP joint | 102 (18), 60-130 | 105 (24), 60-150 |  |  |  |  |

C group: circumferential group / NC group: non-circumferential group / aROM: active range of motion / pROM: passive range of motion /
SD: standard deviation/ °: degree / MCP: metacarpophalangeal joint/ IP: interphalangeal joint
