# Supplementary material for: Six-Strand Flexor Pollicis Longus Tendon Repairs With and Without Circumferential Sutures: A Multicenter Study
Source: Hand (N Y). 2022 Jan 7;18(5):811–9. doi: 10.1177/15589447211057295 (PMC10336814; doi:10.1177/15589447211057295)
Supplement: sj-docx-2-han-10.1177_15589447211057295 – Supplemental material for Six-Strand Flexor Pollicis Longus Tendon Repairs With and Without Circumferential Sutures: A Multicenter Study [file sj-docx-2-han-10.1177_15589447211057295.docx]

| Supplementary Table S2. Strength, DASH and satisfaction scores at weeks 13 and 26 | | | | | |
| --- | --- | --- | --- | --- | --- |
| Examination or test | | Week 13 | | Week 26 | |
|  |  | C group | NC group | C group | NC group |
|  |  | Mean (SD), range | Mean (SD), range | Mean (SD), range | Mean (SD), range |
| Injured thumb | Jamar Hydraulic Hand Dynamometer (kg) | 31 (14), 11-65 | 35 (12), 14-53 | 37 (13), 19-76 | 43 (11), 30-57 |
|  | Jamar Pinch Gauges – key pinch (kg) | 6 (2), 2-10 | 7 (2), 4-9 | 8 (2), 3-13 | 9 (1), 7-11 |
|  | DASH score | 22 (15), 0-55 | 15 (9), 2-26 | 10 (9), 0-25 | 3 (3), 1-8 |
|  | Satisfaction score | 7 (2), 1-10 | 8 (2), 6-10 | 8 (2), 4-10 | 8 (1), 7-10 |
| Un-injured thumb | Jamar Hydraulic Hand Dynamometer (kg) | 38 (12), 22-75 | 43 (11), 25-54 | 41 (13), 20-80 | 46 (12), 28-62 |
|  | Jamar Pinch Gauges – key pinch (kg) | 8 (2), 5-13 | 9 (2), 6-11 | 9 (2), 6-14 | 10 (2), 8-14 |

C Group: circumferential group / NC group: non-circumferential group / DASH: Disability of the Arm, Shoulder, Hand questionnaire /
kg: kilograms / SD: standard deviation
